# Development of Breathable Pebax^®^/PEG Films for Optimization of the Shelf-Life of Fresh Agri-Food Products

**DOI:** 10.3390/membranes11090692

**Published:** 2021-09-07

**Authors:** Thibaut Préfol, Olivier Gain, Guillaume Sudre, Fabrice Gouanvé, Eliane Espuche

**Affiliations:** Ingénierie des Matériaux Polymères, Univ Lyon, Université Lyon 1, CNRS UMR 5223, F-69622 Villeurbanne, France; thibaut.prefol@hotmail.fr (T.P.); olivier.gain@univ-lyon1.fr (O.G.); guillaume.sudre@univ-lyon1.fr (G.S.); fabrice.gouanve@univ-lyon1.fr (F.G.)

**Keywords:** breathable film, CO_2_/O_2_ ideal selectivity, stability, microstructure, hydration properties, thermo-mechanical properties

## Abstract

In this work, thin transparent breathable films were prepared for food packaging applications. The films were obtained by the solvent casting method from both the binary blends Pebax^®^ MH1657 copolymer/ hydroxyl-terminated polyethylene glycol (PEG_OH_) and Pebax^®^ MH1657/polyethylene glycol dimethyl ether (PEG_DME_) as well as the ternary blend Pebax^®^ MH1657/PEG_OH_/PEG_DME_ with a 50/50 and 37.5/62.5 PEG_OH_/PEG_DME_ weight ratio for additive amounts comprised between 0 and 50 wt.%. The microstructure of these materials was investigated by differential scanning calorimetry (DSC) and wide-angle X-ray scattering (WAXS) analyses. Regardless of the PEG’s nature, for a PEG amount inferior to 30 wt.%, the Pebax^®^ and PEG phases were totally miscible. For higher amounts, a phase separation was obtained. In the presence of PEG, a decrease in crystallinity was obtained. The effects of the nature and amount of PEG on the thermo-mechanical, hydration, and gas (CO_2_, O_2_) transport properties were investigated. A study of the film’s stability in terms of composition over time was also performed. From this work, a wide range of films could be proposed with a stable composition over time and adjustable mechanical and gas transport properties for the prolongation of the shelf-life of highly breathable fresh products.

## 1. Introduction

Globalization, development, and the improvement of the living standards within industrialized countries have changed the food diet of our societies over the past few decades. The food we consume increasingly travels the world before it reaches our plates. Therefore, the quality and freshness of products must be preserved throughout the agri-food chain. However, some fresh products, such as fruits and vegetables, are perishable and generally have a short shelf-life [1]. The deterioration of these fresh products starts from the moment when they are separated from the mother plant [1]. Thus, their preservation still constitutes one of the most important challenges.

The metabolic activities of fresh products, including respiration and ripening, continue after their harvest until senescence and death [2,3]. The respiratory activity of a fresh product represents the main factor to consider for the prolongation of its shelf-life [4]. Respiration in plants is the oxidative breakdown of starch, sugars, and organic acids to simpler molecules (e.g., CO_2_, H_2_O) accompanied by a production of energy. Some of this energy is released in the form of heat and some is used as metabolic energy [5,6]. Modified atmosphere packaging (MAP) has been widely used to extend the shelf-life of fresh products by slowing down their respiratory activity and protecting them from the external environment [6,7]. The modified atmosphere is created naturally inside the package due to a dynamic equilibrium between the respiration of the product (i.e., O_2_ uptake and CO_2_ production) and gas transfer through the packaging film. Therefore, the gas transport properties of the packaging film must be well-known and well-suited to the respiration of fresh products in order to obtain a modified atmosphere composition that is beneficial for their preservation [8]. Indeed, for example, an O_2_ concentration that is too low and a CO_2_ concentration that is too high in the package headspace will result in anoxia and fermentative reactions, leading to physiological damage and an acceleration in senescence [9]. Some commodities have elevated respiratory activity. For instance, berries, mushrooms, and broccoli produce a great quantity of CO_2_ (above 250 mL·kg^−1^·day^−1^) [10]. In this case, the packaging film has to be highly permeable to maintain sufficient metabolic activity.

For such applications, micro-perforated and macro-perforated films allowing for a high gas flux have been used [11,12,13]. However, this packaging technology has certain limitations, such as the permselectivity of the perforated package, e.g., the ratio of the CO_2_ permeability coefficient to the O_2_ permeability coefficient, which is near 1 in this case [14]. On the other hand, most dense synthetic polymeric films have a CO_2_/O_2_ permselectivity of around 3–5 [10,15], but they do not exhibit high enough gas permeability levels [16]. Thus, packaging industries are seeking alternatives for agri-food products.

For several years, some poly(ether-*b*-amide) block copolymers, better known under their trade name Pebax^®^, have been recognized as highly permeable. Pebax^®^ is more precisely a multiblock copolymer consisting of linear chains of relatively rigid polyamide (PA) segments interspaced with flexible polyether (PE) segments [17]. The semi-crystalline polyamide moieties—regarded as a very poor permeable phase—impart the mechanical properties to the material, whereas the amorphous polyether moieties act as the main permeable phase due to their high chain mobility [18]. Moreover, the ether linkages, which have a specific affinity for CO_2_ due to quadrupole–dipole interactions, may provide a CO_2_-philic character [19]. The chemical nature of each block of the copolymer may differ. Nylon-6 (PA6), nylon-11 (PA11), and nylon-12 (PA12) are mainly found as amide segments. Concerning the ether segments, polyethylene oxide (PEO), polypropylene oxide (PPO), and polytetramethylene oxide (PTMO) are often used. Thus, by varying the nature, the proportion, and the molar mass of both blocks, a wide range of Pebax^®^ copolymers with very different properties can be obtained. More particularly, Pebax^®^ grades with a high polyether content, such as Pebax^®^ MH1657 (60 polyether wt.%), have been investigated for gas separation applications involving CO_2_ and light gases (such as H_2_, He). Indeed, as the amount of polyether increases, the gas permeability increases [17,18].

The transport of small gas molecules through dense polymer membranes has been successfully described by the solution-diffusion model [20]. Several research works have succeeded in improving the gas transport properties of Pebax^®^ MH1657, in terms of gas permeability and CO_2_/light gas selectivity, by the incorporation of low-molar-mass CO_2_-philic additives [21,22,23]. These small species act as plasticizers when inserted between polymer chains, leading to a decrease in polymer–polymer interactions and an increase in free volume. Consequently, an increase in the gas permeability is obtained [21]. Moreover, increasing the number of polar chemical groups that have a high affinity for CO_2_ improves the CO_2_’s solubility within the material and promotes high CO_2_/light gas selectivity.

For instance, Rabiee et al. investigated the CO_2_, H_2_, N_2_, and CH_4_ transport properties of Pebax^®^ MH1657/glycerol triacetate (GTA) gel membranes. The highest permeability values were obtained for the blend membranes containing 80 wt.% of GTA independently of the tested gases [22]. Indeed, the membrane permeability for CO_2_, H_2_, N_2_, and CH_4_ increased by a factor of 8, 4, 13, and 18, respectively, compared with the neat Pebax^®^ at a pressure of 4 bar and a temperature of 25 °C. On the other hand, the CO_2_/H_2_ selectivity almost doubled. Car et al. prepared and characterized blend membranes comprised of Pebax^®^ MH1657 and hydroxyl-terminated polyethylene glycol with a 200 g·mol^−1^ molar mass (PEG_OH_200). Dense membranes showed excellent compatibility with PEG_OH_200 contents up to 50 wt.% [24]. A CO_2_ permeability of 151 Barrer was reached at 30 °C with a feed pressure of 600 mbar for the membrane comprised of 50 wt.% PEG_OH_200. The CO_2_/N_2_ and CO_2_/CH_4_ selectivity remained unchanged with values around 45 and 15.5, respectively, whereas an enhancement was observed for CO_2_/H_2_ selectivity (from 9 to almost 11) [24]. Yave et al. succeeded in obtaining films with superior CO_2_ separation properties by combining Pebax^®^ MH1657 with polyethylene glycol dimethyl ether (PEG_DME_) [23]. The incorporation of 50 wt.% PEG_DME_ into the Pebax^®^ matrix resulted in an 8-fold increase in the CO_2_ permeability and a simultaneous increase in the CO_2_/H_2_ selectivity from 9.1 to 14.9 [23].

In all these studies, the authors mainly paid attention to CO_2_ and light gases. To the best of our knowledge, no work has been reported in the literature concerning the use of such materials for CO_2_/O_2_ separation with respect to breathable films for packaging applications. Moreover, no study focused on the investigation of possible synergetic effects through the combination of PEG_OH_ and PEG_DME_ in the same membrane. Thus, the objective of the current work is to develop new dense polymeric packaging films able to combine high CO_2_ and O_2_ permeabilities and high CO_2_/O_2_ permselectivity considering both the binary and ternary blends from the Pebax^®^ matrix, PEG_OH_, and PEG_DME_ for a wide range of compositions with the aim of discussing the impact of additive content and the chain end effect as well as the interest in simultaneously using both additives. Moreover, particular attention has been paid to the stability of the developed films over time. Indeed, numerous research works have succeeded in improving Pebax^®^’s transport properties by introducing high amounts of low-molar-mass additives used as plasticizing agents, while no studies have checked on the chemical composition stability of the blended films over time. Indeed, it is well known that, depending on the polymer/additive affinity and the additive amount, exudation phenomena may occur in the presence of low-molar-mass species mobile enough to migrate toward the surface [25,26].

## 2. Materials and Methods

### 2.1. Chemical Compounds

Pebax^®^ MH1657 composed of 40 wt.% of rigid PA6 units and 60 wt.% of flexible PEO units was kindly supplied in pellet form by Arkema (Colombes, France). Hydroxyl-terminated polyethylene glycol with a molar mass of 300 g·mol^−1^ (PEG_OH_) and polyethylene glycol dimethyl ether with a molar mass of 250 g·mol^−1^ (PEG_DME_) were purchased from Sigma Aldrich (Saint Quentin Fallavier, France). The densities of PEG_OH_ and PEG_DME_ are respectively referenced to 1.13 and 1.01 by the supplier. Ethanol (96% purity) was purchased from Sigma Aldrich (Saint Quentin Fallavier, France). All products were used without further purification.

### 2.2. Preparation of the Films

Three series of films were prepared: binary Pebax^®^/PEG_OH_ blends, binary Pebax^®^/PEG_DME_ blends, and ternary Pebax^®^/PEG_OH_/PEG_DME_ blends_._ In all cases, the total amount of additive ranged from 0 to 50 wt.%. Concerning the ternary blends, a 50/50 PEG_OH_/PEG_DME_ weight fraction and a 37.5/62.5 PEG_OH_/PEG_DME_ weight fraction were considered in order to obtain 1/1.2 and 1/2 PEG_OH_/PEG_DME_ molar fractions, respectively. The Pebax^®^/PEG_OH_ and Pebax^®^/PEG_DME_ binary blends were respectively denoted P/PEG_OH_X and P/PEG_DME_X, where X is the amount by weight of the additive in the blend. For instance, the binary blend denoted P/PEG_OH_10 is composed of 90 wt.% of Pebax^®^ and 10 wt.% of PEG_OH_ additive. The ternary blends were denoted P/PEG_OH_X/PEG_DME_Y, where X and Y are the amount by weight of PEG_OH_ and PEG_DME_ in the blend, respectively. For instance, the ternary blend denoted P/PEG_OH_25/PEG_DME_25 is composed of 50 wt.% of Pebax^®^, 25 wt.% of PEG_OH_, and 25 wt.% of PEG_DME_.

Films were prepared by the solvent casting method. Pebax^®^ was dissolved in an ethanol/water (75/25 vol.%) solution under continuous magnetic stirring at 80 °C for 1 h. The polymer concentration was 1.5 wt.%. The desired amount of polyethylene glycol was added after complete dissolution of the Pebax^®^. The solution was stirred for 1 h and then poured into a Teflon ring mold. Solvent evaporation was carried out at room temperature for 48 h. All film characterizations were performed 48 h after the preparation step. For each sample, the thickness (around 50 µm) was determined by averaging ten measurements taken on different parts of the film.

### 2.3. Film Characterization

#### 2.3.1. Differential Scanning Calorimetry (DSC) Measurements

Thermal characteristic parameters (glass transition temperature, melting temperatures, melting enthalpies) were determined using a TA Q200 differential scanning calorimeter (DSC) (TA Instruments, New Castle, DE, USA) equipped with a liquid nitrogen cooling system. DSC experiments were performed on 5–10 mg samples under a helium atmosphere with a heating/cooling/heating cycle comprised between −130 and 250 °C at a standard temperature scan rate of 10 °C·min^−1^. The glass transition temperature (*T*_g_) values were taken at the midpoint of the specific heat capacity (C_p_) curve.

Pebax^®^ is a block copolymer comprised of a polyether phase and a polyamide phase both susceptible to crystallization. It was possible to determine from the DSC thermograms the degree of crystallinity within each phase *i* (polyether or polyamide) according to Equation (1):

(1) $$X_{c,i}\;\;\left(\%\right)=\frac{\Delta H_{m,i}}{w_{i}\cdot \;\Delta H_{m,i}^{\infty }}\times 100$$

where $w_{i}$ is the weight fraction of the considered phase (polyamide or polyether, respectively), $\Delta H_{m,i}$ its melting enthalpy, and $\;\Delta H_{m,i}^{\infty }$ is the melting enthalpy of the respective 100% crystalline polymer ($\Delta H_{mPEO}^{\infty }$ = 166.4 J·g^−1^ and $\;\Delta H_{mPA6}^{\infty }$ = 230 J·g^−1^) [27]. The total degree of crystallinity of the copolymer material can be obtained by considering both phases.

The thermal properties were evaluated during the first heating scan in order to correlate the obtained results to those of transport properties.

#### 2.3.2. Wide Angle X-ray Scattering (WAXS) Analysis

The microstructure of the prepared membranes was characterized at 23 °C using a SAXS/WAXS benchtop beamline Xeuss 2.0 equipped with a Cu anode (*λ* = 1.5406 Å) at the Laboratoire Leon Brillouin (LLB, CEA-Saclay, Gif sur Yvette, France). The distance between the sample and the detector was 17 cm, leading to a *q*-range from 0.1 to 3 Å^−1^. The *q*-calibration was carried out using a silver behenate standard. The samples, which consisted of a stack of 4 pieces of the same film with a total thickness of about 200 µm, were directly placed in the beam path. The signal was detected using a Pilatus 1M detector. The scattering contribution of air was subtracted from the scattering intensity of the samples. The image data treatments took into account the dark images. The intensity was then normalized by the incident flux and the sample thickness. Azimuthal averaging was finally carried out to obtain the profiles of the intensity as a function of *q*. The degree of crystallinity in the total blend was calculated by peak deconvolution using the Origin Pro 2016 software.

#### 2.3.3. Gas Pycnometry

Film density was determined by an AccuPyc II 1340 gas pycnometer (Micromeritics) at 23 °C. The apparatus consisted of two chambers. The first one was filled with helium and the gas was then expanded into the second chamber containing the sample. The volume of gas displaced, as measured by pressure sensors, allowed us to determine the sample volume according to Mariotte’s law. As the sample’s mass was measured beforehand, it was possible to calculate the sample’s apparent density. For better accuracy, a desorption step was performed prior to each measurement. The precision on the density values was estimated to be ±0.01 kg·L^−1^.

#### 2.3.4. Dynamic Mechanical Analysis (DMA)

The thermo-mechanical properties were determined in tensile mode on rectangle-shaped samples (35 × 4.5 mm^2^) using a Q800 dynamic mechanical analyzer (TA Instruments, New Castle, DE, USA). The experiments were performed in the temperature range from −150 to 250 °C with a preloaded force of 0.2 N using a deformation amplitude of 5 µm and a frequency of 3 Hz. The heating rate was fixed at 2 °C·min^−1^.

#### 2.3.5. Thermogravimetric Analysis (TGA)

Thermogravimetric analysis was used to control the film composition by measuring the weight losses associated with the decomposition of each component. The characterizations were performed on 10–20 mg samples with a TA Q500 thermogravimetric analyzer (TA Instruments, New Castle, DE, USA), under a helium atmosphere, from room temperature to 550 °C with a heating rate of 10 °C·min^−1^.

#### 2.3.6. Water Sorption Analysis

Water sorption was investigated for water activities (*a_w_*) between 0.1 and 0.9 by using an Advantage dynamic vapor sorption analyzer (DVS). The experiments were performed at 25 °C on samples with an average mass of 20 mg. Prior to the analysis, a drying step was first performed on the sample by exposure to dry nitrogen. For each water activity level, the water uptake (*M_t_*) was followed as a function of time (*t*) and the water uptake at equilibrium (*M*) was considered to be reached when changes in mass with time were lower than 2 × 10^−4^ mg·min^−1^ for at least 5 consecutive minutes. The water sorption isotherm was plotted from the water uptake at equilibrium (*M* being expressed as g_sorbed water_/g_polymer_) as a function of water activity (*a_w_*).

The diffusion coefficient (*D*) was determined at each water activity level from Equation (2) taking into account the data obtained for *M_t_*/*M* > 0.5 [28]. Indeed, the water uptake was fast and it was not possible to consider with a good accuracy the data for *M_t_*/*M* < 0.5 [28]:

(2) $$\frac{M_{t}}{M}=1-\frac{8}{\pi^{2}}exp\left(\frac{-D\cdot \pi^{2}\cdot t}{L^{2}}\right)$$

$M_{t}$ is the mass of water sorbed at time *t*, M is the mass of water sorbed at equilibrium, and *L* is the sample thickness. *D* was determined from the slope of the straight line obtained from the plot of ln (1 − $M_{t}$*/*M) as a function of *t*.

#### 2.3.7. Gas Permeability

The dioxygen and carbon dioxide permeability coefficients were measured at 23 °C by a constant volume/variable pressure apparatus on films with an effective area of 3 cm^2^. The gas purity was higher than 99%. After a preliminary high vacuum desorption step, gas at a pressure fixed at 1.2 bar was applied in the upstream compartment of the permeation cell. The pressure variation in the downstream compartment was measured as a function of time with a Datametrics pressure sensor (10 Torr). A linear increase in the downstream pressure as a function of time was obtained in the steady state, allowing for the determination of the permeability coefficient (*P*). *P* is expressed in Barrer units (1 Barrer = 10^−10^ cm^3^_STP_·cm·cm^−2^·cm^−1^_Hg_·s^−1^). The uncertainty in the gas permeability coefficient was estimated to be inferior to 10%. The ideal selectivity *α_A/B_* was defined as the ratio of the permeability of two gases A and B, A being more permeable than B:

(3) $$\alpha_{A/B}=\frac{P_{A}}{P_{B}}$$

## 3. Results

**Figure 1:**
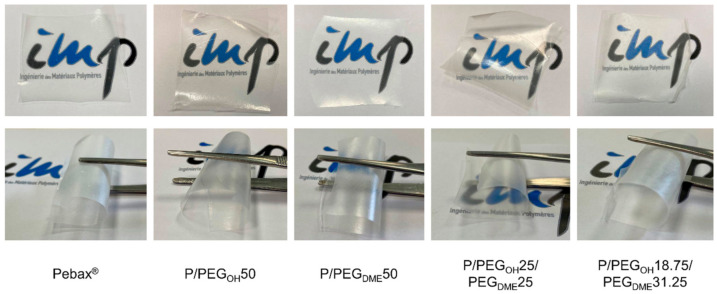
Visual observation of pristine Pebax^®^ and blended Pebax^®^/PEG films with an additive content of 50 wt.% showing their transparency and flexibility properties.

As shown in Figure 1, flexible translucent films were obtained regardless of their composition.

### 3.1. Microstructure Analysis

**Figure 2:**
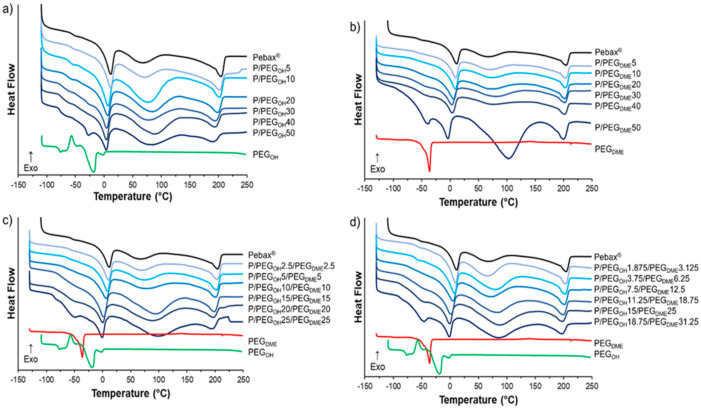
DSC thermograms of the pristine Pebax^®^, PEG additives, and Pebax^®^/PEG_OH_ blends (**a**), Pebax^®^/PEG_DME_ blends (**b**), Pebax^®^/PEG_OH_/PEG_DME_ blends with a 50/50 PEG_OH_/PEG_DME_ weight ratio (**c**), and Pebax^®^/PEG_OH_/PEG_DME_ blends with a 37.5/62.5 PEG_OH_/PEG_DME_ weight ratio (**d**).

Thermal properties of the films composed of binary Pebax^®^/PEGOH and Pebax^®^/PEGDME as well as ternary Pebax^®^/PEGOH/PEGDME blends were studied by DSC analysis with the aim of obtaining information about the miscibility of the PEG additives with the Pebax^®^ matrix. The thermograms corresponding to the first heating run of the blended Pebax^®^/PEG films, the pristine Pebax^®^, and the PEG additives are presented in Figure 2.

The thermogram of the pristine Pebax^®^ exhibits a heat flow (heat capacity) jump at −56 °C corresponding to the glass transition temperature of the soft PE segments. Then, three endothermic peaks were observed. The first peak and the last peak, with values at the peak apex of around 11 °C and 205 °C, were attributed to the melting of the PE and PA crystalline phases, respectively, in agreement with the characteristic separated microphase structure found in block copolymers, such as poly(ether-bloc-amide) [17]. The third broad peak between 50 °C and 125 °C was observed only for the first heating run. It could be attributed to the significant amount of water sorbed during the sample’s storage under ambient conditions due to the presence of polar chemical groups within both Pebax^®^ blocks [29]. It has to be noticed that the glass transition of the PA segments was not detected in the thermograms in agreement with the low amount of PA amorphous phase in the Pebax^®^ MH1657 and the difficulty of determining the glass transition of PA by DSC analysis [17]. The thermogram of PEG_OH_ exhibited a glass transition at −77 °C, followed by a cold crystallization phenomenon from −66 °C to −47 °C and a melting peak centered at −20 °C. For PEG_DME_, the glass transition was observed at −100 °C followed by a melting peak centered at −36 °C. The obtained DSC data for the neat Pebax^®^ and the PEG additives were in good agreement with those reported in the literature [17,23,24,30].

The thermal transitions of the films based on the binary and ternary blends were analyzed with respect to those of Pebax^®^ and PEG references. Regardless of the additive, for additive content inferior to 30 wt.%, the thermograms of all blends were similar to the one obtained for the neat Pebax^®^. No thermal transition due to the presence of the additive was observed. Above 30 wt.% of additive, a melting peak of the additive, which increased as the content of the additive increased, appeared at around −25 and −45 °C for the binary Pebax^®^/PEG_OH_ and Pebax^®^/PEG_DME_ blends, respectively (see Figure 2a,b). Likewise, regarding both ternary blends, a shoulder appeared at around −25 °C corresponding to the melting point of the PEG_DME_ additive (see Figure 2c,d).

**Figure 3:**
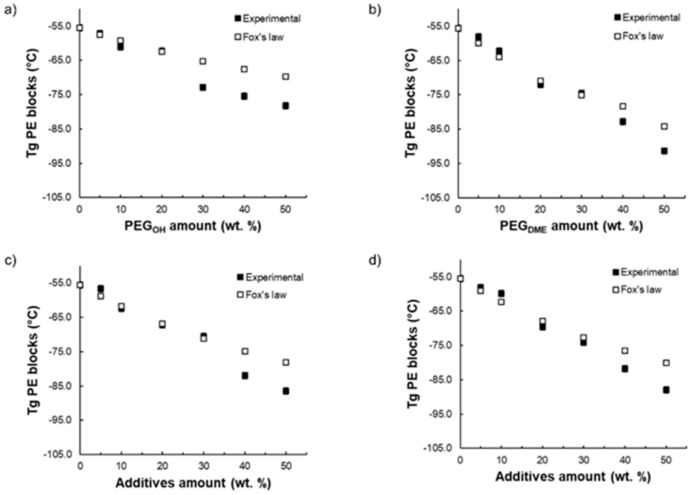
Comparison between the experimental *T*_g_ and the theoretical *T*_g_ calculated by Fox’s law as a function of the additive amount for Pebax^®^/PEG_OH_ blends (**a**), Pebax^®^/PEG_DME_ blends (**b**), Pebax^®^/PEG_OH_/PEG_DME_ blends with a 50/50 PEG_OH_/PEG_DME_ weight ratio (**c**), and Pebax^®^ /PEG_OH_/PEG_DME_ blends with a 37.5/62.5 PEG_OH_/PEG_DME_ weight ratio (**d**).

A partial miscibility of the additives within the copolymer matrix is to be expected since the chemical structures of both PEG additives and polyether blocks are similar. Thus, to confirm the miscibility degree of the PEG additives with the Pebax^®^ soft phase, the experimental *T_g_* values were compared with the glass transition temperature values calculated using Fox’s law (see Figure 3). This law is often used to study the ability of two components to lead to a homogeneous blend:

(4) $$\frac{1}{T_{g}}=\underset{i}{\sum }\frac{w_{i}}{T_{g,i}}$$

where *T_g_* and *T_g,i_* are the glass transition temperature of the blend and the neat components, respectively. $w_{i}$ is the weight fraction of each component *i*. This law has already been used in numerous studies to discuss the miscibility of different additives within Pebax^®^ [31,32].

Figure 3 shows the evolution of the measured *T_g_* of the PE blocks for each blend as a function of the PEG additive amount and compares it to the theoretical *T_g_* calculated from Equation (4). For the binary Pebax^®^/PEG_OH_ and Pebax^®^/PEG_DME_ blends, respectively, a good agreement between the experimental and calculated *T_g_* values was obtained for a PEG_OH_ amount up to 20 wt.% (Figure 3a) and a PEG_DME_ amount up to 30 wt.% (Figure 3b). Thus, in these ranges of composition, the PEG additives were mainly dissolved within the polyether phase of Pebax^®^. For higher additive contents, the experimental and theoretical *T_g_* values differed, showing the occurrence of a phase separation. Surprisingly, the measured *T_g_* values were lower than those calculated from Fox’s law. This result suggests that the *T_g_* values that were evidenced were the signature of the separated rich PEG additive phase. Likewise, for both ternary blends (see Figure 3c,d), a good agreement between the experimental and calculated *T_g_* was obtained for PEG additive amounts up to 30 wt.% suggesting, here again, that the PEG additives were dissolved within the polyether phase of Pebax^®^ in this range of composition. Furthermore, it should be noted that, for the same Pebax^®^/additive ratio, the *T_g_* value was lower when the blend contained a higher PEG_DME_ amount. This result is in agreement with the lower *T_g_* of the PEG_DME_ additive with respect to PEG_OH_ (see Figure 3).

For all binary and ternary blends, it can be observed that the melting temperature of the PE segments (T_m_PE) decreased as the amount of additive increased (see data in the Supplementary Materials). This shift in T_m_PE was more pronounced for the blends containing the PEG_DME_ additive. Indeed, the T_m_PE varied from 11 °C for pristine Pebax^®^ to around 3 °C for Pebax^®^/PEG_OH_, to −3 °C for Pebax^®^/PEG_DME_, and to −1 °C for the ternary Pebax^®^/PEG_OH_/PEG_DME_ blends over the studied composition range. Three reasons could explain this trend: a contribution of the additive melting peak to the melting peak of the polyether block; a decrease in the size of the polyether block’s crystalline lamellas; and the formation of defects within the polyether crystalline domains. It can also be noticed that the enthalpy value related to this melting peak, denoted ∆H_m_PE, first slightly increased for additive contents up to 20 wt.% and then decreased for higher contents (see data in the Supplementary Materials). Thus, at a low PEG content, it seemed that the PEG species could contribute to the melting peak whereas at higher PEG contents, when a specific melting peak of the PEG species was detected, the enthalpy related to the melting peak of PE was mainly governed by the proportion of PE within the Pebax^®^/PEG blend.

**Figure 4:**
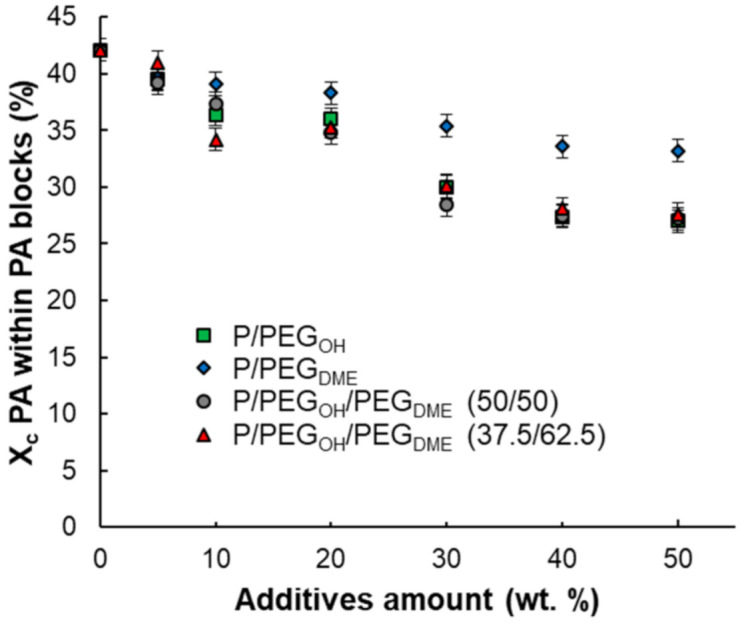
PA crystallinity within PA blocks as a function of additive amount.

Likewise, the melting temperature of the PA blocks (T_m_PA) for all binary and ternary blends decreased with the increase in the additive amount, suggesting that the size of the PA crystalline lamellas decreased or that the formation of defects was favored (see data in the Supplementary Materials). It can also be noted that the melting enthalpy of the polyamide blocks (∆H_m_PA) decreased with the increase in the PEG amount, which is consistent with the fact that the PA proportion decreased in the blend. In order to better assess the impact of the PEG additives on the PA crystallinity, the degree of PA crystallinity within PA blocks was calculated for each formulation and plotted as a function of the additive amount. As shown in Figure 4, for all blends, the PA crystallinity within PA blocks decreased as the additive amount increased, leading to a system with a higher amount of content with an amorphous structure. The degree of PA crystallinity within the PA blocks remained higher for the binary Pebax^®^/PEG_DME_ blends compared with the Pebax^®^/PEG_OH_ blends. Moreover, the degree of PA crystallinity within the PA blocks was similar for the binary Pebax^®^/PEG_OH_ blends and the ternary blends, suggesting that the PEG_OH_ additive played a major role in the ternary blends (see in Figure 4).

**Figure 5:**
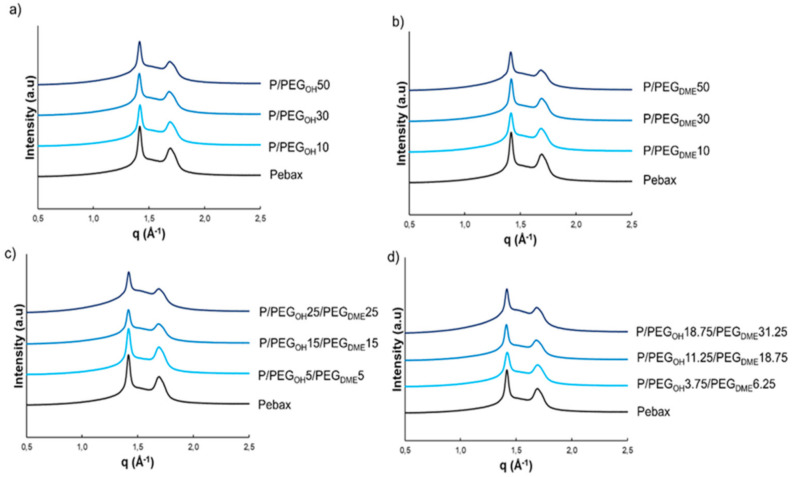
Resulting WAXS patterns of pristine Pebax^®^ and Pebax^®^/PEG blends: Pebax^®^/PEG_OH_ blends (**a**), Pebax^®^/PEG_DME_ blends (**b**), Pebax^®^/PEG_OH_/PEG_DME_ blends with a 50/50 PEG_OH_/PEG_DME_ weight ratio (**c**), and Pebax^®^ /PEG_OH_/PEG_DME_ blends with a 37.5/62.5 PEG_OH_/PEG_DME_ weight ratio (**d**). Curves were shifted vertically for clarity.

WAXS analysis was performed as a complementary method in order to obtain more information about the nature of the crystalline phases present at room temperature in the Pebax^®^ matrix and the binary and ternary blends. Figure 5 displays the WAXS patterns of the studied systems.

WAXS patterns of the pristine Pebax^®^ and of the binary and ternary Pebax^®^-based blends exhibited an amorphous halo and two reflection peaks centered at *q* = 1.41 and 1.69 Å^−1^. These peaks could be assigned to the *α* form (monoclinic) of the PA crystalline phase of the Pebax^®^ copolymer in agreement with the WAXS analysis results obtained on Nylon 6 by Wu and coworkers [33]. Moreover, these two peaks remained at the same *q*-position for all blends, regardless of the nature of the additive or the composition (see Figure 5). No diffraction peak related to a PEO-type crystalline microstructure was observed on WAXS patterns of the pristine Pebax^®^ as well as binary and ternary blends. Indeed, these peaks should be observed at *q* = 1.35 and 1.64 Å^−1^ according to the literature [34,35]. This confirmed that PE segments of Pebax^®^ and PEG additives were in the molten state at 23 °C, which is in accordance with the preliminary results obtained by DSC and the fact that the crystallinity at 23 °C (*X_c_*_,23°C_) resulted only from the PA segments. Moreover, it can be noted that the intensity of the diffraction peaks decreased as the PEG additive amount increased.

*X_c_*_,23°C_ was calculated according to the following equation:

(5) $$X_{c,23{}^{\circ}C\;}\;\left(\%\right)=\frac{A_{c}}{A_{c}+A_{a}}\times 100$$

where *A*_c_ is the area of both peaks and *A_a_* the area attributed to the amorphous halo.

The obtained *X_c_*_,23°C_ values are reported in Table 1 and compared to the values calculated from the DSC experiments, taking into account only the contribution of the PA phase. The *X_c_*_,23°C_ calculated from both WAXS and DSC decreased with the increase in the PEG additive amount. However, it should be noted that there were differences between the degrees of crystallinity obtained by the two methods. Such trends have already been observed in the literature [36] and could be explained by the uncertainty related to the establishment of the amorphous halo for the WAXS analysis and the difficulty of detecting the melting of small crystalline regions using the DSC apparatus, which often lead to an underestimation of the degree of crystallinity determined by DSC with respect to WAXS [37].

Both sets of analysis results reveal that the evolution of *X*_c,23°C_ was in the same order of magnitude as a function of the additive amount, regardless of the nature of the additive.

### 3.2. Density

**Figure 6:**
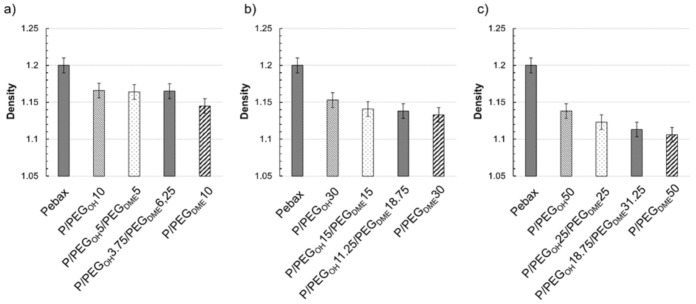
Density values measured for different blend compositions with a total additive amount of 10 wt.% (**a**), 30 wt.% (**b**), and 50 wt.% (**c**).

The evolution of the density as a function of the blend composition is shown in Figure 6 for different total additive contents in the blends (namely 10, 30, and 50 wt.%).

For all binary and ternary blends, the density was lower than those measured on the pristine Pebax^®^. The densities of the ternary blends always ranged between those of the respective binary blends containing the same total additive amount. Moreover, the density decreased with the increase in the PEG additive, which may be indirect evidence of fractional free volume changes within Pebax^®^ [38]. To study more deeply this effect, the experimental density values were compared to a theoretical additive model (Equation (6)):

(6) $$\rho =\underset{i}{\sum }\varphi_{i}\rho_{i}$$

where $\varphi_{i}$ and $\rho_{i}$ are the volume fraction and the densities of the pure components, respectively.

**Figure 7:**
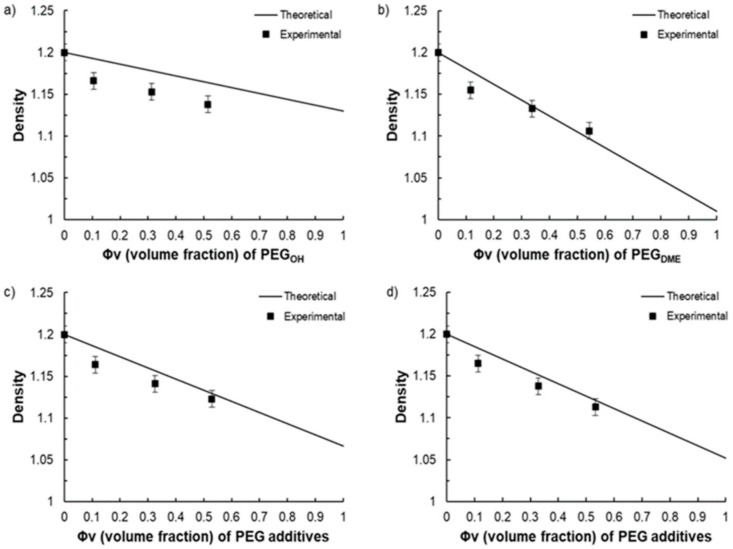
Comparison between the experimental film density measured by helium pycnometry and the theoretical film density calculated by an additive law as a function of the volume fraction of the additive(s) for Pebax^®^/PEG_OH_ blends (**a**), Pebax^®^/PEG_DME_ blends (**b**), Pebax^®^/PEG_OH_/PEG_DME_ with a 50/50 PEG_OH_/PEG_DME_ weight ratio (**c**), and Pebax^®^/PEG_OH_/PEG_DME_ with a 37.5/62.5 PEG_OH_/PEG_DME_ weight ratio (**d**).

As shown in Figure 7a, the Pebax^®^/PEG_OH_ blends displayed lower density values than those predicted by the additive model. This trend was in agreement with that obtained by Car et al. for Pebax^®^/PEG_OH_200 blends [24]. A discrepancy between the experimental and theoretical density values was also observed for both ternary Pebax^®^/PEG_OH_/PEG_DME_ blends and binary Pebax^®^/PEG_DME_ blends (see Figure 7b–d), but the difference between the theoretical and experimental density values decreased as the additive amount increased and totally vanished for the additive amount of 50 wt.%. This discrepancy may have arisen from the fact that the additive model implies that the components retain their own characteristics within the blend, which is not necessarily the case when the components are miscible (in a narrow PEG content range) or when a change in the crystalline Pebax^®^ microstructure (evidenced by a decrease in the crystallinity within the PA phase and a decrease in the PA melting temperature) occurs due to the presence of the additive(s).

### 3.3. Thermal–Mechanical Properties

**Figure 8:**
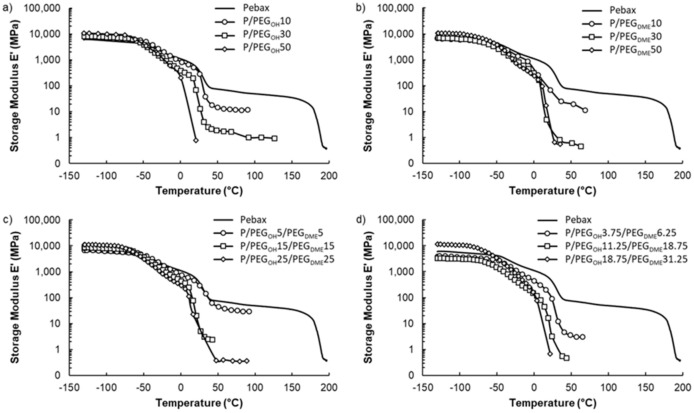
DMA curves showing the thermo-mechanical properties of the pristine Pebax^®^ film, the Pebax^®^/PEG_OH_ films (**a**), the Pebax^®^/PEG_DME_ films (**b**), the Pebax^®^/PEG_OH_/PEG_DME_ films with a 50/50 wt.% PEG_OH_/PEG_DME_ ratio (**c**), and the Pebax^®^/PEG_OH_/PEG_DME_ films with a 37.5/62.5 wt.% PEG_OH_/PEG_DME_ ratio (**d**).

The thermal–mechanical properties of blended Pebax^®^ films were investigated by measuring their temperature-dependent storage modulus (*E’*) in the temperature range from −130 to 200 °C. The evolution of *E’* as a function of temperature is displayed in Figure 8 for each studied system.

Four domains were visible on the storage modulus curve of the pristine Pebax^®^ film. For temperatures inferior to −50 °C, the Pebax^®^ copolymer was in the glassy state. From −50 °C, the significant gain in mobility of the amorphous polyether segments resulted in a sharp decrease in the storage modulus, which was due to the alpha transition of the PE block of the copolymer. Thereafter, an even more pronounced second decrease in *E’* was observed between 15 °C and 40 °C, which was attributed to the melting of the polyether crystalline phases. Finally, a plateau only depending on the rigid polyamide phase of the Pebax^®^ was observed until the PA crystalline phase melted. This thermo-mechanical behavior is typical of the Pebax^®^ materials family [39].

For all studied film series, the thermo-mechanical properties evolved in a similar way as a function of the additive amount, independently of the PEG chain end’s nature. Indeed, the storage modulus values measured on the glassy plateau (between −130 and around −50 °C) were of the same order of magnitude (around 9 GPa) and did not vary significantly as a function of the additive structure and amount. However, a shift in the first drop in the storage modulus towards lower temperatures occurred as the PEG content increased. This behavior was in agreement with the decrease in the glass transition temperature of the polyether blocks evidenced by the DSC analysis and can be explained by the plasticization of the Pebax^®^ polyether chains. The second sharp decrease in the storage modulus was also shifted toward lower temperatures with the PEG amount. This behavior can be assigned to the decrease in the melting temperature of the polyether crystalline phases as shown by DSC. Finally, it has to be noticed that the storage modulus values at the plateau (above 50 °C) were considerably decreased with the PEG amount. This result can be attributed to the presence of these low-molar-mass additives, which have poor mechanical properties, and also to the decrease in the amide phase proportion in the total blend, which contributes to the mechanical resistance properties of the film. As a consequence, the plateau range was narrowed. It was even no longer observable for high additive contents due to the complete collapse of the film. Thus, adding a PEG amount above 30 wt.% appeared to be detrimental to the mechanical properties of the films regardless of the nature of the PEG chain ends.

### 3.4. Hydration Properties

**Figure 9:**
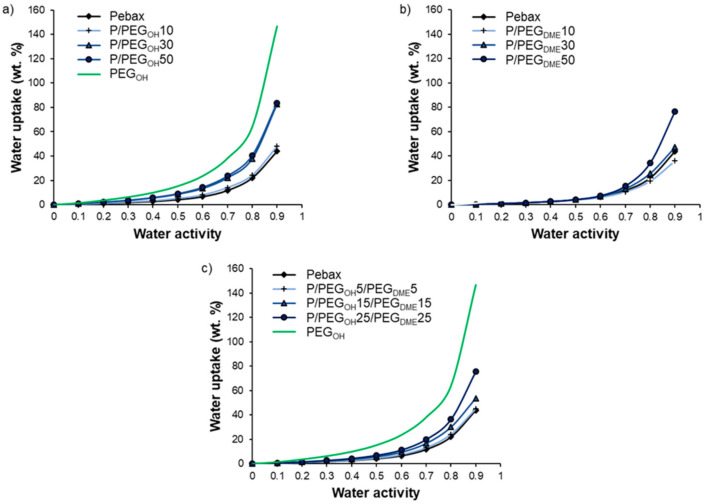
Water vapor sorption isotherms of Pebax^®^/PEG_OH_ films (**a**), Pebax^®^/PEG_DME_ films (**b**), and Pebax^®^/PEG_OH_/PEG_DME_ films with a 50/50 wt.% PEG_OH_/PEG_DME_ ratio (**c**).

Water vapor sorption properties were investigated for three different systems through a thermodynamic analysis (sorption isotherms) and a kinetic analysis (diffusion coefficients). Figure 9 displays the water vapor sorption isotherms of Pebax^®^/PEG_OH_ blends (Figure 9a), Pebax^®^/PEG_DME_ blends (Figure 9b), and Pebax^®^/PEG_OH_/PEG_DME_ blends with a 50/50 PEG_OH_/PEG_DME_ weight ratio (Figure 9c). The water sorption isotherms of the pristine Pebax^®^ and the PEG_OH_ additive are reported as references. The water uptake values for different activities are detailed in Table 2.

First of all, the isotherm curve of Pebax^®^ had a BET III shape according to the classification of Brunauer, Emmett, and Teller [40]. A similar isotherm shape has been observed for other Pebax^®^-type copolymers [32]. The water uptake was important at a low water activity level (4% for an activity level of 0.5) due to the presence of ether and amide polar groups in the Pebax^®^ structure. Moreover, the chain flexibility allowed for important swelling that led to an important increase in the water uptake at a high activity level (44% for an activity level of 0.9). The isotherm curve of PEG_OH_ also had a BET III shape with even higher water uptake values (146.7% for an activity level of 0.9). Not surprisingly, the isotherm curves of binary and ternary systems had a BET III shape.

**Figure 10:**
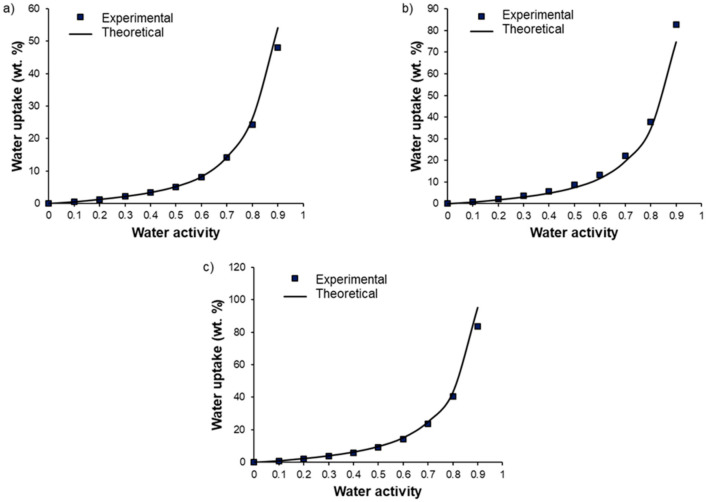
Comparison between experimental water uptake values and theoretical water uptake values calculated by an additive model as a function of water activity for Pebax^®^/PEG_OH_ blends with a PEG_OH_ amount of 10 wt.% (**a**), 30 wt.% (**b**), and 50 wt.% (**c**).

For Pebax^®^/PEG_OH_ blends, the water uptake values were higher than those obtained for the pristine Pebax^®^, especially at high activity levels (*a_w_* > 0.5). Moreover, a shift in the water isotherm towards higher water uptake values was observed as the PEG_OH_ amount increased (see Figure 9a). This behavior can be explained by the hydrophilic character of PEG_OH_ and the plasticizing effect of the additive. The experimental water uptake values determined at different activities for each blend composition (90/10, 70/30, and 50/50 wt.%) were then compared to theoretical values calculated by an additive model (see Figure 10):

(7) $$M=w_{Pebax}\cdot M_{Pebax}+w_{PEG_{OH}}\cdot M_{PEG_{OH}}\;$$

where M, $M_{Pebax}$, and $M_{PEG_{OH}}$ are the water uptake values of the considered blend, Pebax^®^ and PEG_OH_, respectively, for the same activity level. $w_{Pebax}$ and $w_{PEG_{OH}}$ are the weight fraction of Pebax^®^ and PEG_OH_ in the blend, respectively.

In all cases, the experimental isotherms exhibited very close water uptake values compared to those calculated by the additive model, showing that each component made its own contribution to the water uptake.

For Pebax^®^/PEG_DME_ blends, no significant difference was observed in the water uptake values compared to those obtained for neat Pebax^®^ up to an activity level of 0.5. The water uptake then increased with the PEG_DME_ amount, showing that the plasticization provided by the PEG_DME_ additive played a key role in water sorption at high activity levels (see Figure 9b). Moreover, it should be noted that the water uptake values obtained for Pebax^®^/PEG_DME_ blends were lower than those obtained with the Pebax^®^/PEG_OH_ blends in the whole range of water activities for the same PEG amount. This can be explained by the more hydrophilic character of the chain ends of PEG_OH_.

**Figure 11:**
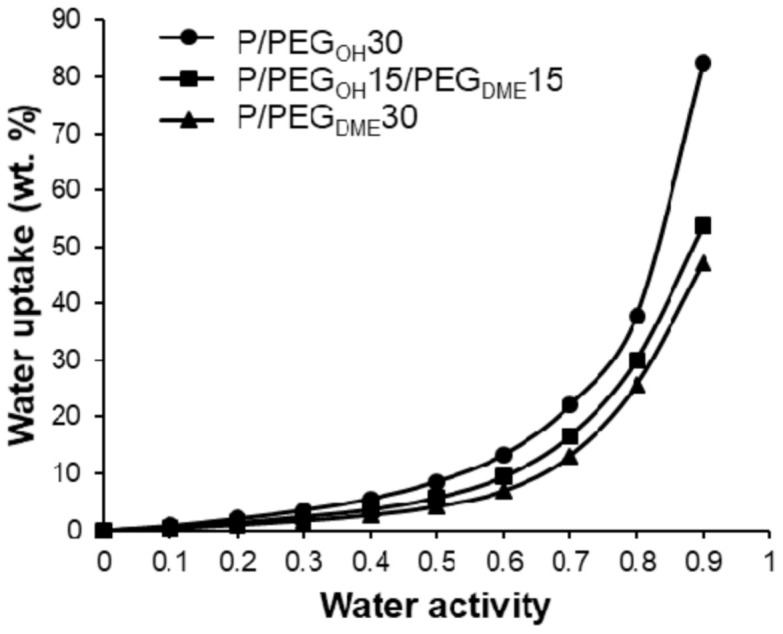
Comparison of the evolution of water uptake as a function of activity for Pebax^®^/PEG_OH_ blends (circle), Pebax^®^/PEG_OH_/PEG_DME_ blends (square), and Pebax^®^/PEG_DME_ blends (triangle) with a comparable PEG amount of 30 wt.%.

For the ternary Pebax^®^/PEG_OH_/PEG_DME_ blends with a 50/50 PEG_OH_/PEG_DME_ weight ratio, the water uptake values ranged between those measured on the binary systems in the whole range of water activities as shown in Figure 11.

**Figure 12:**
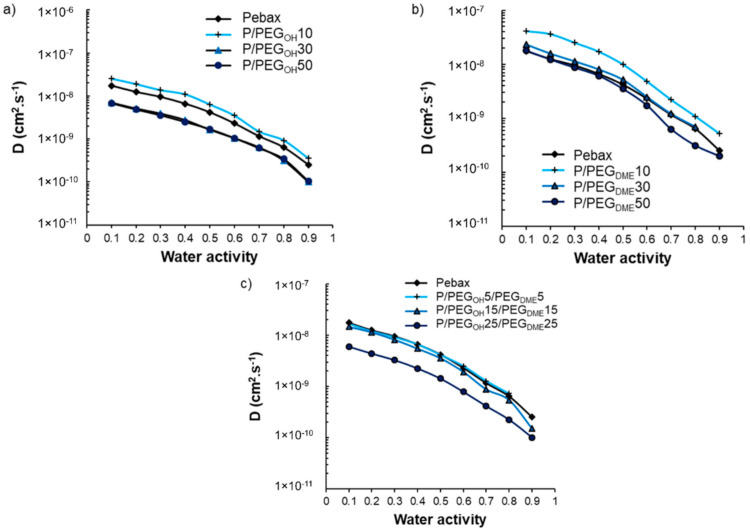
Evolution of the diffusion coefficient as a function of the film composition and water activity for Pebax^®^/PEG_OH_ blends (**a**), Pebax^®^/PEG_DME_ blends (**b**), and Pebax^®^/PEG_OH_/PEG_DME_ blends with a 50/50 wt.% PEG_OH_/PEG_DME_ ratio (**c**).

The impact of PEG additives on the water sorption kinetics was also investigated. The water diffusion coefficients (*D*) were determined after having checked that the water uptake curves obeyed a Fickian diffusion mechanism. The evolution of *D* as a function of water activity is displayed in Figure 12.

As one can observe in Figure 12, all curves presented the same shape, consisting of a slow-down of the water diffusion as the water activity increased. This trend is in agreement with a BET-III-type sorption mechanism and the formation of water aggregates at high activity levels [40,41]. For all studied film series, the evolution of the water diffusion as a function of the PEG additive amount was similar and not monotonic. Indeed, the values of the kinetic parameter increased as the PEG amount increased from 0 to 10 wt.% and then decreased and became similar or lower than the values obtained on the pristine Pebax^®^ for blends comprising additive amounts of 30 and 50 wt.%.

As discussed previously, the PEG additives were totally dissolved within the polyether phase of Pebax^®^ at a low PEG amount (10 wt.%), which led to a decrease in water–Pebax^®^ as well as water–PEG interactions, making the water’s diffusion easier. However, a new PEG-rich phase was formed at high PEG amounts (30 and 50 wt.%) in which strong water–PEG interactions could be established due to the polar nature of PEG, thus explaining the decrease in the diffusion coefficient. A similar trend was also observed for Pebax^®^/ionic liquid blends [32].

The antiplasticization phenomenon observed at activities above 0.5 was more deeply analyzed using the following equation:

(8) $$D=D_{agg}\cdot e^{-\beta M}$$

where *D_agg_* is the limit diffusion aggregation coefficient at nil concentration, *β* is the antiplasticization coefficient, and *M* is the water uptake in the material. The antiplasticization coefficient *β* was determined for each film from the linear regression of the decreasing part of the curve ln(*D*) = f(*M*). *β* values are summarized in Table 2. The *β* of the pristine Pebax^®^ and the binary as well as ternary Pebax^®^/PEG systems was very similar with values comprised between 3 and 5, showing that the addition of PEG species within the Pebax^®^ matrix did not significantly modify the impact of the water clustering mechanism at high activities.

### 3.5. Gas Transport Properties

The CO_2_ and O_2_ permeability coefficients as well as the ideal CO_2_/O_2_ selectivity were determined on the pristine Pebax^®^ and the different Pebax^®^/PEG film series (Table 3). The experimental values of permeability measured on the pristine Pebax^®^ MH1657 are in accordance with those reported in the literature [42,43]. As a reminder, according to the DSC and WAXS results, the flexible polyether blocks of the Pebax^®^ copolymer were totally amorphous at the temperature of the permeation experiments (23 °C), whereas the rigid polyamide blocks were semi-crystalline. Therefore, gas transport was mainly governed by the polyether phase under these conditions. As one can observe from the permeability values reported in Table 3, the CO_2_ permeability coefficient of the pristine Pebax^®^ was considerably higher than the one measured for O_2_ (84 and 3.9 Barrer, respectively). Thus, the pristine Pebax^®^ film possessed a high CO_2_/O_2_ selectivity of 21.5, which could be assigned to the presence of CO_2_-philic ether groups within the copolymer that increase the CO_2_ solubility [19,44].

**Figure 13:**
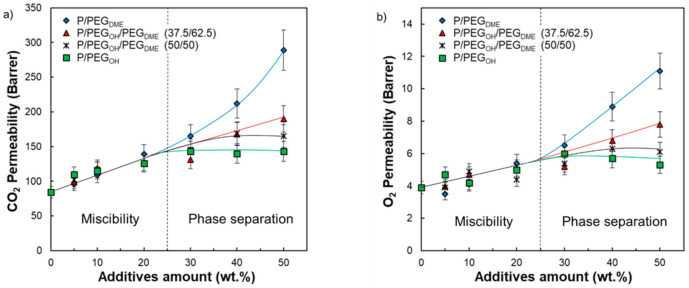
Evolution of the CO_2_ permeability coefficient (**a**) and the O_2_ permeability coefficient (**b**) as a function of the additive amount for each Pebax^®^/PEG blend.

The impact of PEG addition on the gas transport properties of Pebax^®^ was investigated for the binary and ternary blends. The data presented in Table 3 and Figure 13 clearly show that all blended Pebax^®^/PEG films exhibited superior CO_2_ and O_2_ permeability coefficients and higher ideal CO_2_/O_2_ selectivity than the pristine Pebax^®^. We have shown that the PEG additives led to a plasticizing effect that increased as the additive amount increased. Moreover, a decrease in density and a decrease in the PA blocks’ crystallinity was evidenced upon PEG addition. All these changes are favorable to an increase in gas diffusion, explaining the general increase in gas permeability. Moreover, the presence of CO_2_-philic groups within both Pebax^®^ and PEG additives should contribute to high CO_2_ solubility and therefore to a reinforcement of selectivity.

Looking more carefully at the evolution of the CO_2_ and O_2_ permeability coefficients as a function of the system’s composition, two domains were distinguished (see Figure 13). In the first domain (for an additive amount inferior to 30 wt.%), the CO_2_ and O_2_ permeability coefficients increased and remained quite similar for the same additive content regardless of its nature. It is worth noting that the composition domain ranging from 0 to 30 wt.% of additives corresponded to the miscibility domain between Pebax^®^ and the PEG additives. Then, it seems that, in this domain, the effect of PEG chain ends was negligible. For additive contents above 30 wt.%, different behaviors were observed depending on the chain ends of the PEG additives (Figure 13).

For Pebax^®^/PEG_OH_ blends, the CO_2_ and O_2_ permeability coefficients reached a plateau around 143 and 5.5 Barrer, respectively. Thus, the CO_2_ and O_2_ permeability coefficients were improved by around 70% and 40%, respectively, compared with the neat Pebax^®^ matrix. It is worth noting that the plateau reached for the experimental CO_2_ permeability coefficients corresponds to the intrinsic CO_2_ permeability coefficient of a completely amorphous hydroxyl-terminated PEO precisely determined to be 143 Barrer by Lin and Freeman [19]. Thus, the transport properties seemed to be limited by the intrinsic permeability of the most permeable component (PEG_OH_ in this case).

The CO_2_ and O_2_ permeability coefficients measured on Pebax^®^/PEG_DME_ films with a PEG_DME_ amount superior to 30 wt.% were higher than those obtained for Pebax^®^/PEG_OH_ blends for a similar additive amount. Indeed, both the CO_2_ and O_2_ permeability coefficients did not reach a plateau value but steadily increased up to 289 and 11.1 Barrer for a PEG_DME_ amount of 50 wt.%, respectively (see Figure 13). Thus, the CO_2_ and O_2_ permeability coefficients were improved by a factor of 3 compared with the pristine Pebax^®^. The observed deviation in the gas transport properties between Pebax^®^/PEG_OH_ and Pebax^®^/PEG_DME_ blends can only be explained by a chain end effect. Indeed, the presence of a bulky methyl end group probably hinders hydrogen-bonding interactions between polymer chains and allows for a higher free volume available for the transport of small gas molecules [23]. It has to be noticed that, to the best of our knowledge, the intrinsic CO_2_ permeability of a completely amorphous PEG_DME_ has not been referenced in the literature and so could not be compared to the one of a completely amorphous hydroxyl-terminated PEO.

The CO_2_ and O_2_ permeability coefficients measured on both ternary Pebax^®^/PEG_DME_/PEG_OH_ blends ranged between those of Pebax^®^/PEG_OH_ and Pebax^®^/PEG_DME_ blends for PEG amounts superior to 30 wt.%. Higher gas permeability coefficients were obtained for ternary blends having the highest PEG_DME_ proportion. Indeed, the P/PEG_OH_18.75/PEG_DME_31.25 blend had CO_2_ and O_2_ permeability coefficients of 190 and 7.8 Barrer, respectively, with respect to the P/PEG_OH_25/PEG_DME_25 blend, which had CO_2_ and O_2_ permeability coefficients of 169 and 6.3 Barrer, respectively. Moreover, it seemed that the molar fraction of PEG_DME_/PEG_OH_ within the blend needed to exceed the ratio 1/1 in order to avoid a plateau effect for the studied range of compositions. Thus, the PEG_DME_ additive was particularly interesting to introduce into the blend in order to increase the gas permeability coefficients.

For the three studied series, an average increase in the ideal CO_2_/O_2_ selectivity of between 10% and 20% was also observed regardless of the film’s composition (Table 3). This increase in both permeability and selectivity is not common for polymer materials. Indeed, a natural intrinsic trade-off exists between these parameters for dense polymeric films as described by Robeson via the establishment of an upper bound for numerous gas pairs [45,46]. For our systems, the increase in CO_2_/O_2_ selectivity could be attributed to the increase in the proportion of CO_2_-philic ether groups within the material. Therefore, the developed Pebax^®^/PEG films have high potential as breathable films for food packaging applications as a result of their interesting gas transport properties. Among them, the Pebax^®^/PEG_DME_ films with high PEG_DME_ contents offer the best gas transport properties.

### 3.6. Film Stability

**Figure 14:**
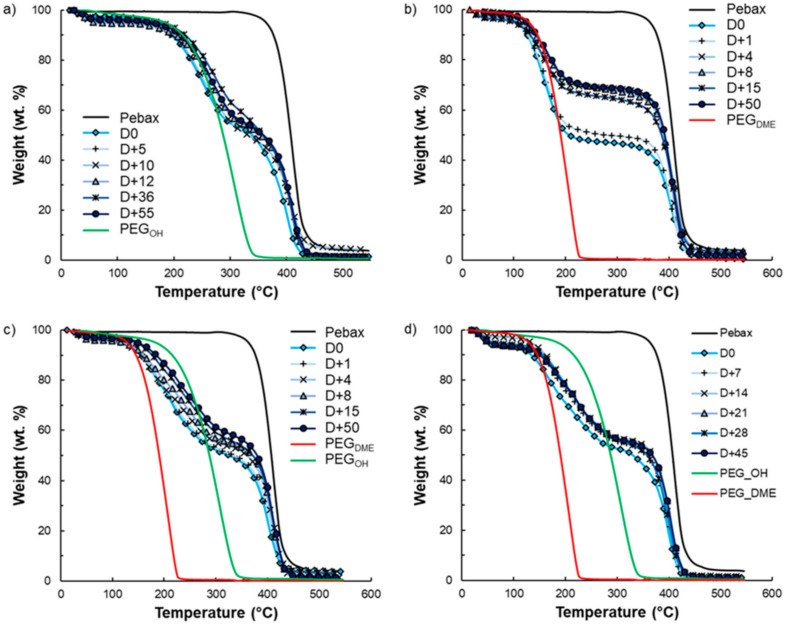
Film stability over time followed by TGA analysis for Pebax^®^/PEG_OH_ blends (**a**), Pebax^®^/PEG_DME_ blends (**b**), Pebax^®^/PEG_OH_/PEG_DME_ blends with a 50/50 PEG_OH_/PEG_DME_ weight ratio (**c**), and Pebax^®^/PEG_OH_/PEG_DME_ blends with a 37.5/62.5 PEG_OH_/PEG_DME_ weight ratio (**d**). The additive amount was 50 wt.% for all systems. “D0” represents the day when a dry film was obtained, i.e., 48 h after the film’s conception.

Film stability is an important criterion for long-term use. It is known that exudation phenomena may occur following the addition of low-molar-mass additives to a polymer matrix depending on the polymer–additive interactions and the additive amounts. That is why film stability in terms of composition over time was investigated by TGA analysis for the highest amount of additive studied in this work. TGA curves of the pristine Pebax^®^, the PEG_OH_ and PEG_DME_ additives, and binary and ternary Pebax^®^/PEG blends with an additive amount of 50 wt.% are displayed in Figure 14. During heating from room temperature to 550 °C, degradation in one step occurred between 350 and 500 °C for pristine Pebax^®^. For PEG additives, an average weight loss of 4 wt.% was observed between 50 and 100 °C, which was attributed to water evaporation, followed by total weight loss between 150 °C and 350 °C for PEG_OH_ and between 100 and 250 °C for PEG_DME_.

Thus, the degradation temperatures of the pure components (Pebax^®^ vs. PEG additives) can be clearly distinguished, allowing us to follow the composition of the binary films over time as shown in Figure 12b and Figure 14a. Indeed, weight loss in two steps was observed on each binary Pebax^®^/PEG blend due to the degradation of the additive and the Pebax^®^, in agreement with the results obtained on pure components. Concerning the ternary blends, the degradation temperatures of PEG_OH_ and PEG_DME_ were too close to differentiate their signatures on the TGA curves. However, the first instance of weight loss observed in the temperature range 100–350 °C could clearly be assigned to the additives’ degradation, whereas the weight loss at a higher temperature (around 400 °C) was the signature of the Pebax^®^’s degradation (see Figure 14c,d). It is also worth noting that an average water loss of 4 wt.% was also observed on the TGA curves of all Pebax^®^/PEG blends below 100 °C.

**Figure 15:**
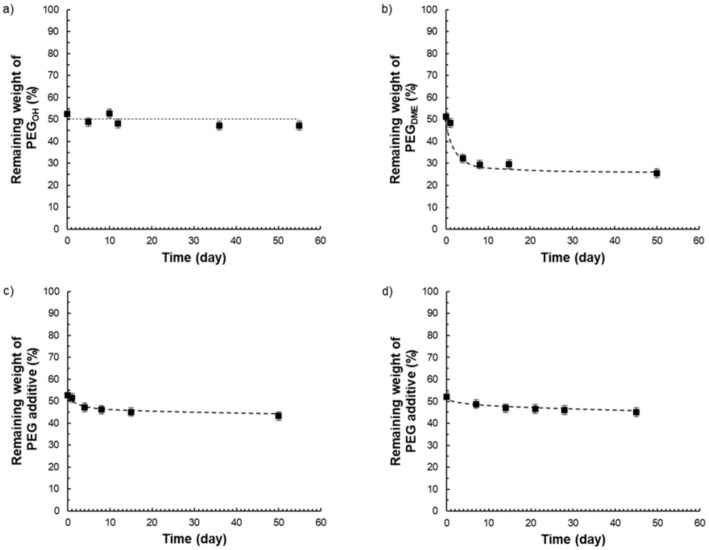
Weight of the remaining PEG additive within a film initially containing a PEG amount of 50 wt.% as a function of time for Pebax^®^/PEG_OH_ blends (**a**), Pebax^®^/PEG_DME_ blends (**b**), Pebax^®^/PEG_OH_/PEG_DME_ blends with a 50/50 PEG_OH_/PEG_DME_ weight ratio (**c**), and Pebax^®^/PEG_OH_/PEG_DME_ blends with a 37.5/62.5 PEG_OH_/PEG_DME_ weight ratio (**d**).

Thus, from the different TGA curves, it was possible to determine the amount of additive that remained in the films after different storage times under atmospheric conditions (23 °C, 30% RH) (see Figure 15).

Pebax^®^/PEG_OH_ films with a PEG_OH_ amount of 50 wt.% were stable in composition over time (Figure 15a), whereas Pebax^®^/PEG_DME_ films containing an additive amount of 50 wt.% were not stable in composition over time as illustrated in Figure 15b. For this latter system, the matrix/additive proportion evolved from a 50/50 weight ratio to a constant 75/25 weight ratio after 50 days due to the exudation of the PEG_DME_ additive. Further studies carried out on Pebax^®^/PEG_DME_ films with additive amounts of 30 and 40 wt.% confirmed a stabilization of the film composition towards a 75/25 matrix/PEG_DME_ weight ratio after a testing period of 50 days (Supplementary Materials). For the ternary blend P/PEG_OH_25/PEG_DME_25, a slight weight loss (lower than 6%) was observed after 50 days (see Figure 15c). Likewise, a slight weight loss (lower than 6%) was obtained after 45 days for the ternary blend P/PEG_OH_18.75/PEG_DME_31.25 (Figure 15d). From the results obtained on binary blends, it could be supposed that the weight losses observed on both ternary blends could be assigned to the PEG_DME_’s exudation.

## 4. Conclusions

In this study, transparent films were prepared by the solvent casting method from two binary Pebax^®^/PEG_OH_ and Pebax^®^/PEG_DME_ blends and two ternary Pebax^®^/PEG_OH_/PEG_DME_ blends with a 50/50 and 37.5/62.5 PEG_OH_/PEG_DME_ weight ratio. The additive amounts ranged between 5 and 50 wt.%. For all studied films and additive amounts inferior to 30 wt.%, the PEG additives were mainly dissolved in the Pebax^®^ polyether phase. However, phase separation occurred for additive amounts superior to 30 wt.%. The analysis of the storage modulus values in the temperature range from −150 to 200 °C showed that the thermo-mechanical properties decreased as the PEG amount increased due to the incorporation of low-molar-mass additive(s) with poor mechanical properties and the decrease in the proportion of rigid polyamide blocks. However, both binary and ternary blends with a PEG additive amount of 30 wt.% maintained a sufficient mechanical strength up to 50 °C. The water sorption mechanism in terms of kinetic and thermodynamic behaviors was studied following PEG addition. For all systems, a BET-III-type isotherm was obtained. The water uptake increased as the additive amounts increased in accordance with the additivity law. However, a non-monotonic evolution of the kinetic parameter was observed as a function of the PEG amount. At a low additive amount (10 wt.%), the dissolution of PEG species within the polyether phase of Pebax^®^ led to a decrease in water–Pebax^®^ as well as water–PEG interactions, making the water’s diffusion easier. At high PEG amounts (30 and 50 wt.%), the formation of a PEG-rich phase and strong water–PEG interactions could be established, leading to a decrease in the diffusion coefficient. All Pebax^®^/PEG films exhibited enhanced gas transport properties in comparison with the pristine Pebax^®^. The increase in CO_2_ and O_2_ permeability coefficients with the additive amounts was attributed to the plasticizing effect of the PEG additive(s) (leading to a gain in mobility of the polyether segments), the increase in the free volume, and the decrease in the amide phase’s crystallinity in the total blend. Moreover, two domains were distinguished on the curves of gas (CO_2_ and O_2_) permeability as a function of the PEG additive amount. For additive amounts inferior to 30 wt.%, which corresponded to the miscibility domain, the CO_2_ and O_2_ permeability coefficients increased but did not depend on the type of chain end of the additive. On the other hand, above 30 wt.% of additive, e.g., in the composition range in which phase separation occurs, the permeability highly depended on the additive’s chemical structure. A plateau was observed for PEG_OH_-based blends, corresponding to the intrinsic permeability of a completely amorphous hydroxyl-terminated PEO. A continuous permeability increase was obtained with Pebax^®^/PEG_DME_ blends. Pebax^®^/PEG_DME_ films exhibited the highest gas permeability coefficients, probably due to the presence of bulky methyl end groups that can hinder hydrogen-bonding interactions between polymer chains and allow for a higher free volume to be made available for the transport of small gas molecules. All ternary Pebax^®^/PEG_OH_/PEG_DME_ blends exhibited functional properties intermediate to those obtained for both Pebax^®^/PEG_OH_ and Pebax^®^/PEG_DME_ binary blends. For all studied film series, the increase in the CO_2_ permeability coefficients was higher than that of the O_2_ permeability coefficients following PEG addition, due to the addition of CO_2_-philic ether groups, leading to an average improvement in the ideal CO_2_/O_2_ selectivity of around 20%. Finally, exudation phenomena were observed on films containing PEG_DME_ amounts superior to 25 wt.%, whereas Pebax^®^/PEG_OH_ films were stable in the whole range of compositions (0–50 wt.%), showing once again the influence of the end chains of the additive. Therefore, different films with a stable composition over time and adjusted mechanical and gas transport properties could be selected from among the wide range of films developed in this work as viable alternatives for breathable food packaging applications related to the preservation of fresh products.

## Figures and Tables

**Table 1:** Comparison of the crystallinity degree at 23 °C determined by DSC and WAXS analysis (the standard deviation on *Xc* values is greater than 2%).

Films	*X_c_*_,23°C_ (%)	*X_c_*_,23°C_ (%)
	DSC	WAXS
Pebax^®^	17	25
P/PEG_OH_10	13	23
P/PEG_OH_30	8	20
P/PEG_OH_50	6	18
P/PEG_DME_10	14	22
P/PEG_DME_30	10	18
P/PEG_DME_50	7	15
P/PEG_OH_5/PEG_DME_5	14	25
P/PEG_OH_15/PEG_DME_15	8	18
P/PEG_OH_25/PEG_DME_25	6	16
P/PEG_OH_3.75/PEG_DME_6.25	12	23
P/PEG_OH_11.25/PEG_DME_18.75	8	21
P/PEG_OH_18.75/PEG_DME_31.25	6	16

**Table 2:** Water uptake for different activities (0.1, 0.3, 0.5, 0.7, and 0.9) and the antiplasticization coefficient of Pebax^®^/PEG_OH_ blends, Pebax^®^/PEG_DME_ blends, and Pebax^®^/PEG_OH_/PEG_DME_ blends with a 50/50 wt.% PEG_OH_/PEG_DME_ ratio.

	Water Uptake (%)					*β* (g_sample_/g_water_)
	*a*_w_ = 0.1	*a*_w_ = 0.3	*a*_w_ = 0.5	*a*_w_ = 0.7	*a*_w_ = 0.9	
Pebax^®^	0.41	1.72	4.11	11.69	43.84	5.5
PEG_OH_	1.4	6.3	15.3	38.1	146.7	
P/PEG_OH_10	0.48	2.2	5.13	14.1	48.08	5.2
P/PEG_OH_30	0.82	3.56	8.62	22.11	82.61	3.3
P/PEG_OH_50	0.82	3.71	9.09	23.63	83.77	3.2
P/PEG_DME_10	0.41	1.71	4.09	12.07	46.33	3.8
P/PEG_DME_30	0.41	1.72	4.21	13.12	47.28	3.0
P/PEG_DME_50	0.39	1.68	4.30	15.32	76.72	2.7
P/PEG_OH_5/PEG_DME_5	0.44	1.91	4.55	13.22	45.04	2.9
P/PEG_OH_15/PEG_DME_15	0.53	2.43	5.74	16.67	53.77	5.4
P/PEG_OH_25/PEG_DME_25	0.58	2.69	6.86	19.85	75.53	3.0

**Table 3:** CO_2_ and O_2_ permeability coefficients and ideal CO_2_/O_2_ selectivity of the pristine Pebax^®^, the Pebax^®^/PEG_OH_ films, the Pebax^®^/PEG_DME_ films, and the Pebax^®^/PEG_OH_/PEG_DME_ films (the standard deviation is greater than 10% for the permeability values and greater than 20% for the selectivity values).

Films	Permeability (Barrer)		Ideal Selectivity
	CO_2_	O_2_	CO_2_/O_2_
Pebax^®^	84	3.9	21.5
P/PEG_OH_5	110	4.7	23.4
P/PEG_OH_10	115	4.2	27.4
P/PEG_OH_20	126	5.0	25.2
P/PEG_OH_30	143	6.0	23.8
P/PEG_OH_40	140	5.7	24.6
P/PEG_OH_50	143	5.3	27.0
P/PEG_DME_5	96	3.5	27.4
P/PEG_DME_10	109	4.1	26.6
P/PEG_DME_20	139	5.4	25.7
P/PEG_DME_30	165	6.5	25.4
P/PEG_DME_40	212	8.9	23.8
P/PEG_DME_50	289	11.1	26.0
P/PEG_OH_2.5/PEG_DME_2.5	102	3.9	26.2
P/PEG_OH_5/PEG_DME_5	117	4.9	23.9
P/PEG_OH_10/PEG_DME_10	126	4.4	28.6
P/PEG_OH_15/PEG_DME_15	146	5.4	27.0
P/PEG_OH_20/PEG_DME_20	169	6.3	26.8
P/PEG_OH_25/PEG_DME_25	165	6.1	27.0
P/PEG_OH_1.875/PEG_DME_3.125	99	4.0	24.8
P/PEG_OH_3.75/PEG_DME_6.25	119	4.7	25.3
P/PEG_OH_7.5/PEG_DME_12.5	128	5.1	25.1
P/PEG_OH_11.25/PEG_DME_18.75	131	5.2	25.2
P/PEG_OH_15/PEG_DME_25	168	6.8	24.7
P/PEG_OH_18.75/PEG_DME_31.25	190	7.8	24.4

## Data Availability

Data are available on request.

## Supplementary Materials

The following are available online at https://www.mdpi.com/article/10.3390/membranes11090692/s1, Table S1: Thermal properties of pristine Pebax and binary blends, Table S2: Thermal properties of pristine Pebax and ternary blends.
