# Supplementary material for: Development of Breathable Pebax®/PEG Films for Optimization of the Shelf-Life of Fresh Agri-Food Products
Source: Membranes (Basel). 2021 Sep 7;11(9):692. doi: 10.3390/membranes11090692 (PMC8470709; doi:10.3390/membranes11090692)
Supplement: Supplementary file 1 [file membranes-11-00692-s001.zip › membranes-1340691-supplementary.pdf]

Supporting Information

Development of Breathable Pebax®/PEG Films for Optimization of the Shelf-Life of Fresh Agri-Food Products

Thibaut Préfol, Olivier Gain, Guillaume Sudre, Fabrice Gouanvé and Eliane Espuche *

Univ Lyon, Université Lyon 1, CNRS UMR 5223, Ingénierie des Matériaux Polymères, F-69622 Villeurbanne, France; thibaut.prefol@hotmail.fr (T.P.); olivier.gain@univ-lyon1.fr (O.G.); guillaume.sudre@univ-lyon1.fr (G.S.); fabrice.gouanve@univ-lyon1.fr (F.G.)

* Correspondence: eliane.espuche@univ-lyon1.fr

S1: Thermal properties of pristine Pebax® and binary Pebax®/PEG blends.

Films	T_g (° C)	T_m PEG_{DME} (° C)	T_m PEG_{OH} (° C)	T_m PE (° C)	T_m PA (° C)	ΔH_m PE (J/g_{sample})	ΔH_m PA (J/g_{sample})	X_c PA within PA blocks (%)
Pebax®	-55.6	-	-	11.3	204.7	24.1	38.7	42.1
$P/PEG_{OH}5$	-57.2	-	-	11.2	201.8	26.7	34.5	39.5
$P/PEG_{OH}10$	-61.1	-	-	7.2	202.1	28.4	30.1	36.4
$P/PEG_{OH}20$	-62.3	-	-	6.4	198.6	31.4	26.5	36.0
$P/PEG_{OH}30$	-72.9	-	-25.2	4.6	194.3	21.2	19.3	30.0
$P/PEG_{OH}40$	-75.5	-	-26.9	4.3	193.7	22.8	15.1	27.4
$P/PEG_{OH}50$	-78.2	-	-28.2	3.2	190.3	13.8	12.4	27.0
$P/PEG_{DME}5$	-58.1	-	-	10.1	204.2	28.7	34.6	39.6
$P/PEG_{DME}10$	-62.3	-	-	9.3	203.8	32.2	32.4	39.1
$P/PEG_{DME}20$	-72.0	-	-	3.6	203.0	30.7	28.2	38.3
$P/PEG_{DME}30$	-74.6	-43.2	-	2.5	202.3	25.4	23.8	35.4
$P/PEG_{DME}40$	-82.8	-46.0	-	-0.3	201.3	19.2	20.7	33.6
$P/PEG_{DME}50$	-91.4	-42.3	-	-3.3	200.6	14.2	16.5	33.2

S2: Thermal properties of ternary Pebax®/PEG blends.

Films	T_g (° C)	T_m PEG_{DME} (° C)	T_m PEG_{OH} (° C)	T_m PE (° C)	T_m PA (° C)	ΔH_m PE (J/g_{sample})	ΔH_m PA (J/g_{sample})	X_c PA within PA blocks (%)
P/PEG_{OH}2.5/PEG_{DME}2.5	-56.7	-	-	10.2	204.2	27.3	34.3	39.2
P/PEG_{OH}5/PEG_{DME}5	-62.3	-	-	8.6	202.6	30.5	31.0	37.4
P/PEG_{OH}10/PEG_{DME}10	-67.2	-	-	6.4	201.0	35.0	25.6	34.8
P/PEG_{OH}15/PEG_{DME}15	-70.6	-44.0	*	1.1	198.7	32.4	18.3	28.4
P/PEG_{OH}20/PEG_{DME}20	-81.9	-45.8	*	-0.4	197.2	24.4	15.2	27.5
P/PEG_{OH}25/PEG_{DME}25	-86.5	-47.5	-	-1.2	195.5	20.9	12.5	27.2
P/PEG_{OH}1.875/PEG_{DME}3.125	-58.2	-	-	9.4	203.7	25.8	35.8	41.0
P/PEG_{OH}3.75/PEG_{DME}6.25	-59.8	-	-	7.7	203.0	28.5	28.3	34.2
P/PEG_{OH}7.5/PEG_{DME}12.5	-69.6	-	-	5.8	201.5	33.3	26.0	35.3
P/PEG_{OH}11.25/PEG_{DME}18.75	-74.1	-47.2	-	1.7	199.7	27.2	19.4	30.1
P/PEG_{OH}15/PEG_{DME}25	-81.8	-42.9	-	-0.8	198.0	20.0	15.5	28.1
P/PEG_{OH}18.75/PEG_{DME}31.25	-87.9	-44.7	-	-1.0	197.0	16.6	12.7	27.6
